# Experimental Investigation on Stability, Viscosity, and Electrical Conductivity of Water-Based Hybrid Nanofluid of MWCNT-Fe_2_O_3_

**DOI:** 10.3390/nano11010136

**Published:** 2021-01-08

**Authors:** Solomon O. Giwa, Mohsen Sharifpur, Mohammad H. Ahmadi, S. M. Sohel Murshed, Josua P. Meyer

**Affiliations:** 1 Department of Mechanical Engineering, Olabisi Onabanjo University, Ibogun 112104, Nigeria; sologiwa2002@yahoo.com; 2 Department of Mechanical and Aeronautical Engineering, University of Pretoria, Pretoria 0002, South Africa; josua.meyer@up.ac.za; 3 Department of Mechanical Engineering, University of Science and Culture, Tehran 1461968151, Iran; 4 Faculty of Mechanical Engineering, Shahrood University of Technology, Shahrood 3619995161, Iran; mohammadhosein.ahmadi@gmail.com; 5 Center for Innovation, Technology and Policy Research (IN+), Department of Mechanical Engineering, Instituto Superior Técnico, University of Lisbon, 1049-001 Lisbon, Portugal

**Keywords:** nanofluids, Fe_2_O_3_ nanoparticle, multiwalled carbon nanotubes, viscosity, electrical conductivity, hybrid nanofluids

## Abstract

The superiority of nanofluid over conventional working fluid has been well researched and proven. Newest on the horizon is the hybrid nanofluid currently being examined due to its improved thermal properties. This paper examined the viscosity and electrical conductivity of deionized water (DIW)-based multiwalled carbon nanotube (MWCNT)-Fe_2_O_3_ (20:80) nanofluids at temperatures and volume concentrations ranging from 15 °C to 55 °C and 0.1–1.5%, respectively. The morphology of the suspended hybrid nanofluids was characterized using a transmission electron microscope, and the stability was monitored using visual inspection, UV–visible, and viscosity-checking techniques. With the aid of a viscometer and electrical conductivity meter, the viscosity and electrical conductivity of the hybrid nanofluids were determined, respectively. The MWCNT-Fe_2_O_3_/DIW nanofluids were found to be stable and well suspended. Both the electrical conductivity and viscosity of the hybrid nanofluids were augmented with respect to increasing volume concentration. In contrast, the temperature rise was noticed to diminish the viscosity of the nanofluids, but it enhanced electrical conductivity. Maximum increments of 35.7% and 1676.4% were obtained for the viscosity and electrical conductivity of the hybrid nanofluids, respectively, when compared with the base fluid. The obtained results were observed to agree with previous studies in the literature. After fitting the obtained experimental data, high accuracy was achieved with the formulated correlations for estimating the electrical conductivity and viscosity. The examined hybrid nanofluid was noticed to possess a lesser viscosity in comparison with the mono-particle nanofluid of Fe_2_O_3_/water, which was good for engineering applications as the pumping power would be reduced.

## 1. Introduction

The exceptional thermal and flow properties exhibited by nanofluid compared with those of conventional thermal transporting media have projected this special fluid as a subject of intense global research. Early studies in this context have measured the viscosity and thermal conductivity of nanofluids with diverse base fluids (ethylene glycol (EG), water, propylene glycol, glycerol, etc.) and found that these properties of the nanofluids were enhanced in relation to the base fluids [1,2,3,4,5,6,7,8,9,10]. However, underlying mechanisms for such enhancements of these properties of nanofluids, particularly for thermal conductivity, are not yet explored, and there are also controversies and inconsistencies in literature results [10,11,12,13]. Outside the viscosity and thermal conductivity, the electrical conductivity (EC), specific heat capacity, dielectric, and density of various nanofluids prepared from different nanoparticles (Cu, MgO, CuO, CNT, SiO_2_, ZnO, TiO_2_, Al_2_O_3_, Fe_2_O_3_, Fe_3_O_4_, spinels etc.) and dispersed in several base fluids (water, propylene glycol, ethylene glycol, bio-glycol, palm oil, glycerol, ionic fluid, coconut oil, engine oil, etc.) have been subsequently examined at various mass/volume concentrations or fractions for different temperature ranges [14,15,16,17,18,19,20].

Hybridization of different types of nanoparticles to prepare hybrid nanofluids was first investigated by Jana and co-workers [21]. The idea behind this was to augment the thermal conductivity of nanofluid over that of conventional fluids. Jana and co-workers [21] synthesized aqueous carbon nanotubes (CNTs), Cu and Au nanofluids, and Cu-CNT and Au-CNT nanofluids and measured their thermal conductivity. They noticed that the hybrid nanofluids yielded a lower thermal conductivity relative to the mono-particle nanofluids. Suresh et al. [22] examined the thermal conductivity and viscosity of water-based Al_2_O_3_-Cu (90:10) nanofluids with varying volume concentrations (0.1–2%) at ambient temperature. The enhancement of the thermal conductivity by 1.47–12.11% and viscosity by 8–115%, in comparison with water, was reported. Both properties were noticed to improve as the volume concentration increased. Conflicting with the result of Jana et al. [21], Suresh et al. [22] showed that hybrid nanofluids have higher thermal conductivity than mono-particle nanofluids (Al_2_O_3_/water). Similarly, Chen et al. [23], who used water-based Ag-MWCNT nanofluid, reported lower thermal conductivity for water-based MWCNT nanofluid when compared with the hybrid nanofluid. The thermal conductivity of the water-based multiwalled CNT and γ-Al_2_O_3_ (at an equal weight ratio (1:1)) nanofluids for different volume concentrations (0–1%) at the room temperature was examined [24]. A maximum enhancement of 20.68% was achieved with 1 vol%. 

Esfe et al. [25] used an equal volume concentration of water-based mono-particle nanofluids (Al_2_O_3_ and MWCNTs) to formulate water-based Al_2_O_3_-MWCNT nanofluids in an effort to measure the thermal conductivity at temperatures of 303–323 K. They noticed that the thermal conductivity was augmented when temperature and volume concentration increased in comparison with water. The rheological study of engine oil (EO)-based hybrid nanofluids (Al_2_O_3_-MWCNTs (75:25%)) was studied under varying shear rates (1333–13,333 s^−1^), temperatures (25–50 °C), and volume concentrations (0–1%) by Dardan et al. [26]. The hybrid nanofluids were noticed to exhibit Newtonian behaviors. The viscosity improved with volume concentration increase and reduced with temperature rise. The highest enhancement of 46% was observed with 1 vol%. Afrand and co-workers [27] measured the viscosity of EO-based SiO_2_-MWCNT hybrid nanofluids (at equal volumes of nanoparticles) under varying temperatures (25–60 °C) and volume concentrations (0.0625–1%). The hybrid nanofluids showed an enhancement of viscosity as the volume concentrations increased. The hybrid nanofluids were observed to be higher than those of SiO_2_/EO and MWCNT/EO nanofluids with a maximum enhancement of 37.4% for 1 vol% at 60 °C.

In the work of Megatif et al. [28], equal weights of CNT-TiO_2_ nanoparticles (0.1, 0.15, and 2.0 wt%) were suspended in water to synthesize the hybrid nanofluids, and the thermal conductivity, density, viscosity, and specific heat capacity were determined at 25–40 °C. These properties were found to be improved for the hybrid nanofluids compared to the mono-particle CNT nanofluids. The viscosity, specific heat capacity, and density of the hybrid nanofluids diminished with rising temperature, whereas the thermal conductivity followed the reverse trend. The rheological behavior of MWCNT-SiO_2_ (50:50 vol%)/EG-water (50:50 vol%) nanofluids was examined under varying shear rates (0.612–122.3 s^−1^), volume concentrations (0.0625–2%), and temperatures of 27.5–50 °C [29]. The nanofluids examined were found to demonstrate shear-thinning flow as the power-law index was below unity. Recently, Kakavandi and Akbari [30] studied the thermal conductivity of EG-water-based MWCNT-SiC/EG using a similar base fluid and ratio of nanoparticles as the work of Eshgarf and Afrand [29] with concentrations of 0–0.75 vol% and temperatures of 25–50 °C. The thermal conductivity was improved by 33% at 0.75 vol% and 50 °C when compared with water-EG. 

Esfe et al. [31] experimentally determined the thermal conductivity of DWCNT-ZnO/EG (10:90) with concentration and temperature ranges of 0.045–1.9 vol% and 30–50 °C, respectively. At 50 °C and 1.9 vol%, the thermal conductivity was augmented by 24.9%. They revealed that the addition of 10% DWCNT nanoparticles to 90% ZnO nanoparticles to formulate the hybrid nanofluids caused the thermal conductivity of EG-based ZnO nanofluids to be enhanced. Additionally, their cost analysis showed that it was more economical to use hybrid nanofluids than mono-particle nanofluids. Moldoveanu et al. [32] studied the thermal conductivity of aqueous mono-particle (TiO_2_ and Al_2_O_3_ for 1–3 vol%) and hybrid (Al_2_O_3_ (0.05 vol%)-TiO_2_ (0.05–2.5 vol%) nanofluids at temperatures of 20 to 50 °C. The measured property was enhanced by 10.7–14.1%, 8.5–17.7%, and 15.3–19.2% for aqueous TiO_2_, Al_2_O_3_, and Al_2_O_3_-TiO_2_ nanofluids, respectively. An investigation into the impact of variation in the volume concentration (0–2.3%) and temperature (25–50 °C) on the thermal conductivity of EG-based hybrid nanofluids of functionalized MWCNT-Fe_3_O_4_ (at equal volumes) was carried out by Harandi et al. [33]. The thermal conductivity of the hybrid nanofluid was augmented by 30% at 50 °C and 2.3 vol%. The rheological behavior of an identical hybrid nanofluid (at equal amounts) and temperature range like that of Harandi et al. [33] was performed using shear rates of 12.24–73.44 s^−1^ and volume concentration of 0.1–1.8% [34]. The result showed that the nanofluids exhibited Newtonian behavior for volume concentrations of 0.1–0.8% and non-Newtonian flow for the nanofluids beyond 0.8 vol%. 

Shi et al. [35] investigated the thermophysical properties (viscosity, thermal conductivity, and specific thermal capacity) of Fe_3_O_4_ and Fe_3_O_4_-MWCNT nanofluids with 0.25 vol%. They reported higher thermal conductivity and viscosity and lower specific heat capacity for Fe_3_O_4_-MWCNT nanofluid compared to Fe_3_O_4_. The introduction of MWCNT particles to formulate the hybrid nanofluid was observed to enhance the thermal conductivity and viscosity but attenuated the specific heat capacity as the Fe_3_O_4_ nanoparticles possess a better specific heat capacity than the MWCNT particles. Recently, a study on the thermal properties (viscosity, density, surface tension, specific heat capacity, and thermal conductivity) of three-nanocomponent water-based hybrid nanofluids was conducted by Mousavi et al. [36]. Hybrid nanofluids of CuO-MgO-TiO_2_/deionized water (DIW) were formulated in five different mixture ratios with volume concentrations of 0.1–0.5 vol%, and the thermal properties were measured at temperatures of 15–60 °C. The results showed that the hybrid nanofluid with a mixture ratio of 60:30:10 (CuO-MgO-TiO_2_) was the best as it had the lowest viscosity (36.4% for 0.5 vol% at 60 °C), highest thermal conductivity (78.6% for 0.1 vol% at 15 °C), and lowest surface tension when compared with DIW. Goodarzi et al. [37] investigated the behavior and viscosity of EO-based ZnO-MWCNT (25:75) nanofluids under changing shear rates (666.5–13,300 s^−1^), temperatures (5–55 °C), and volume concentrations (0.05–0.8%). They reported a Newtonian flow for all the samples and at the studied temperatures. A temperature rise was observed to reduce the viscosity, whereas increasing the concentration improved the viscosity of the hybrid nanofluids, with an enhancement of 5–20%. 

The above literature survey supports the fact that the hybridization of nanoparticles engaged in formulating hybrid nanofluids yielded an improvement in their thermal properties. However, there are limited studies in the open literature regarding the stability and the thermal properties of hybrid ferrofluids. In addition, there is a scarcity of documentation on the thermophysical properties of MWCNT nanoparticle-based ferrofluids (F_3_O_4_, Fe_2_O_3_, Co_3_O_4_, etc.) at different mixing ratios of bi-nanoparticles. This present study involved an experimental measurement of the electrical conductivity and viscosity of MWCNT-Fe_2_O_3_/DIW nanofluids with a bi-nanoparticle mixing ratio of 80:20 (weight % basis) for volume concentrations and temperatures ranging from 0.05% to 1.5% and 15 °C to 55 °C, respectively. Furthermore, research progress in this context revealed that there is a notable dearth of knowledge on the electrical conductivity of hybrid nanofluids in the public domain. 

## 2. Methodology

### 2.1. Materials

Nanoparticles of γ-Fe_2_O_3_ and functionalised MWCNTs were used in this work. The γ-Fe_2_O_3_ nanoparticles (20–30 nm diameter as specified by the manufacturer) were sourced from Nanostructured and Amorphous Materials Inc., TX, USA, while the f-MWCNT nanoparticles (length: 10–30 μm; outer diameter: 10–20 nm and inner diameter: 3–5 nm) were purchased from MKnano Company, ON, Canada. Sodium dodecyl sulphate (SDS) with the purity of ≥98.5% bought from Sigma-Aldrich, Berlin, Germany was used as a surfactant to affect the stability of the studied hybrid nanofluids. The thermal properties of the base fluid and nanoparticles used in this study are provided in Table 1. It is noted that some properties and parameters of nanomaterials are provided in the company’s product datasheets.

### 2.2. Equipment

When suspending the bi-nanoparticles in the deionized (DIW) water, a sonicator (Hielscher, Germany) was employed to homogenize the mixture. A programmable water bath (LAUDA ECO RE1225, Berlin, Germany) was deployed to keep the mixture at a low temperature during sonication and used to keep the nanofluids at the specific temperatures while measuring the thermophysical properties. Other pieces of equipment, such as a digital weighing balance (Radwag AS 220.R2 (Radom, Poland) with an accuracy of ±0.01 g), pH meter (Jenway 3510, Staffordshire, UK; –2 to 19.999 range with ±0.003 accuracy), vibro-viscometer (SV-10; A&D, Tokyo, Japan; with ±3% accuracy), UV–visible spectrophotometer (Jenway, Staffordshire, UK), transmission electron microscope (JEOL JEM-2100F, Tokyo, Japan)—dry sample type, and electrical conductivity meter (EUTECH Instrument (Singapore) with ±1% accuracy), were used for various purposes in this work. 

### 2.3. Hybrid Nanofluid Preparation and Stability

In the formulation of the hybrid nanofluids (Fe_2_O_3_ (80%) and MWCNTs (20%)), a two-step method was used. To ensure proper stability, the pH and electrical conductivity of the formulated MWCNT-Fe_2_O_3_/DIW nanofluids were monitored while SDS amounts and sonication parameters (amplitude, frequency, and sonication time) were optimized at a 0.1% volume concentration and room temperature (20 °C). SDS to bi-nanoparticle weight ratios of 0.4–1.0 were examined. After obtaining the optimum values of amplitude, frequency, sonicating time, and the ratio of SDS to hybrid nanoparticle weight (dispersion fraction), hybrid nanofluids of various volume concentrations (0.1–1.5%) were formulated according to Equation (1).

(1) $$\varphi =\left(\frac{X_{Fe_{2}O_{3}}{\left(\frac{M}{\rho }\right)}_{Fe_{2}O_{3}}+X_{MWCNT}{\left(\frac{M}{\rho }\right)}_{MWCNT}}{X_{Fe_{2}O_{3}}{\left(\frac{M}{\rho }\right)}_{Fe_{2}O_{3}}+X_{MWCNT}{\left(\frac{M}{\rho }\right)}_{MWCNT}+{\left(\frac{M}{\rho }\right)}_{DIW}}\right)$$

where *X* = ratio of each nanoparticle type; *M* = weight of material; *ρ* = density of material. 

The morphology and dispersion of the bi-particles in the hybrid nanofluids were monitored using TEM. Viscosity, UV–visible spectrophotometry, and visual inspection methods were employed to monitor the nanofluids’ stability. The nanofluid can be considered stable if one of the properties has not changed during the period of stability check. However, for our case, both the UV–visible spectrophotometry and viscosity techniques were carried out for 43 h while the visual inspection was done at weekly intervals for a month.

### 2.4. Electrical Conductivity and pH

The electrical conductivity meter was first calibrated with the use of standard calibration fluid supplied by the manufacturer. On calibration, the standard fluid was measured at 25 °C (in triplicates) and an average of 1413.6 μS·cm^−1^ was recorded. Thereafter, the electrical conductivity of DIW and hybrid nanofluids was determined at temperatures of 15–55 °C (at 10 °C intervals). Buffer solutions were used to calibrate the pH meter at a pH values of 4, 7, and 10. Thereafter, the measurement of pH values of the hybrid nanofluids was conducted. The uncertainty of the electrical conductivity and pH measurements was 6.2% and 0.04%, respectively. In comparison with DIW, the relative electrical conductivity and the improvement recorded for MWCNT-Fe_2_O_3_/DIW nanofluids were evaluated as expressed in Equations (2) and (3), respectively.

(2) $$\sigma_{rel}=\frac{\sigma_{hnf}}{\sigma_{bf}}$$

(3) $$\sigma_{enhan}\left(\%\right)=\left(\frac{\sigma_{hnf}-\sigma_{bf}}{\sigma_{bf}}\right)\times 100$$

where

*σ_hnf_* = measured electrical conductivity (hybrid nanofluids) and
*σ_bf_* = measured electrical conductivity (DIW).

### 2.5. Viscosity

Calibration of the vibro-viscometer was carried out prior to viscosity measurement of the DIW and MWCNT-Fe_2_O_3_/DIW nanofluids at temperatures of 15 °C to 55 °C. A percent error of 1.6% was estimated when the measured viscosity of DIW was compared to the standard viscosity of water. The uncertainty of the viscosity measurement was 7.02%. Relative viscosity and improvement of the viscosity of MWCNT-Fe_2_O_3_/DIW nanofluids compared with DIW were evaluated using Equations (4) and (5), respectively.

(4) $$\mu_{rel}=\frac{\mu_{hnf}}{\mu_{bf}}$$

(5) $$\mu_{enhan}\left(\%\right)=\left(\frac{\mu_{hnf}-\mu_{bf}}{\mu_{bf}}\right)\times 100$$

where

*μ_hnf_* = measured viscosity of hybrid nanofluids and
*μ_bf_* = measured viscosity of DIW.

The expression in Equation (6) was used to evaluate the margin of deviation (MOD) of the thermal properties of MWCNT-Fe_2_O_3_/DIW nanofluids.

(6) $$MOD\left(\%\right)=\left(\frac{V_{Exp.}-V_{Pred.}}{V_{Exp.}}\times 100\right)$$

where

*V_Exp._* = experimental data and
*V_Pred._* = predicted data.

It is worth stating that the measured variable (Z), temperature (*T*), weight of hybrid nanoparticles (*m*), and volume of DIW were sources of error associated with the measurement of pH, μ, and σ. The errors were propagated using Equation (7) to estimate the uncertainty related to pH, μ, and σ.

(7) $$U\left(\%\right)=\pm \sqrt{{\left(\frac{∆Z}{Z}\right)}^{2}+{\left(\frac{∆T}{T}\right)}^{2}+{\left(\frac{∆m}{m}\right)}^{2}+{\left(\frac{∆V}{V}\right)}^{2}}$$

## 3. Results and Discussion

### 3.1. Preparation of Hybrid Nanofluids

**Figure 1:**
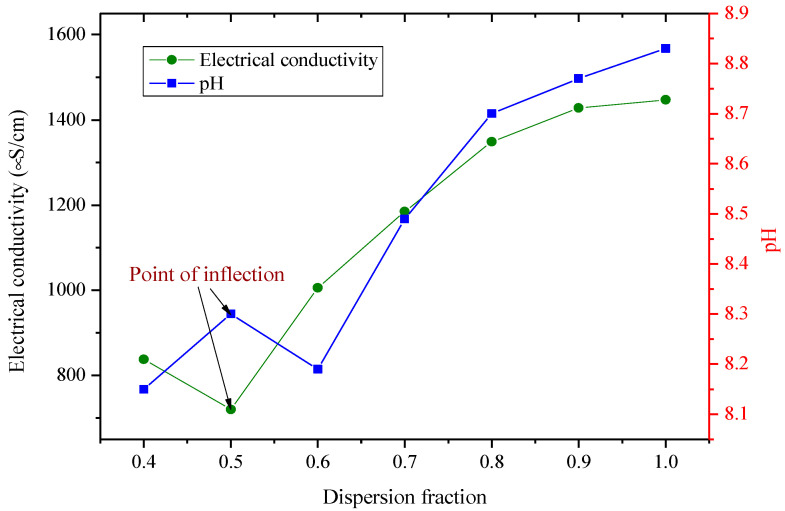
Optimum dispersion fraction of sodium dodecyl sulphate (SDS) in hybrid nanofluids via electrical conductivity and pH monitoring.

**Figure 2:**
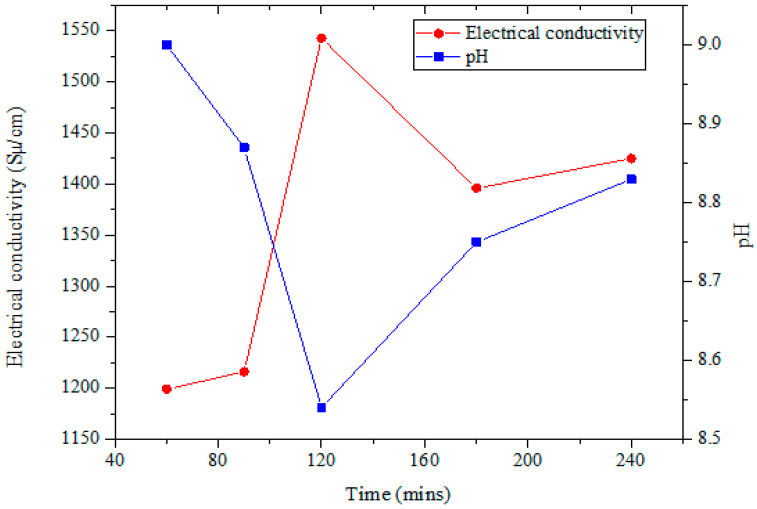
Optimum sonication time of hybrid nanofluids via electrical conductivity and pH monitoring.

The preparation of stable hybrid nanofluids is a very vital step prior to their characterization and thermophysical property measurement, hence the need to optimize several parameters toward achieving this. The electrical conductivity has been reported as a viable property that can be employed to achieve the critical micelle concentration (CMC) of the surfactant utilized for nanofluid preparation [41,42]. The point of inflection of this property is known to be the CMC. In this study, the optimum ratio of SDS to hybrid nanoparticle weight (dispersion fraction), sonicator amplitude, and sonicating time were determined through the monitoring of the pH and electrical conductivity of MWCNT-Fe_2_O_3_/DIW nanofluids (0.1 vol%) at room temperature, as presented in Figure 1 and Figure 2. A turning point (inflection) was observed for the pH and electrical conductivity at an optimum dispersion fraction of 0.5 (Figure 1). Again, the point of inflection was noticed at 120 min for the electrical conductivity in determining the optimum sonication time (Figure 2). The optimum sonication time also indicated a turning point for pH, leading to the optimum (lowest) pH for the MWCNT-Fe_2_O_3_/DIW nanofluid. In this present study, the hybrid nanofluids (at all volume concentrations) were prepared by sonicating at an amplitude of 70%, frequency of 70%, and sonication time of 120 min using a dispersion fraction of 0.5.

### 3.2. Stability of Hybrid Nanofluid

**Figure 3:**
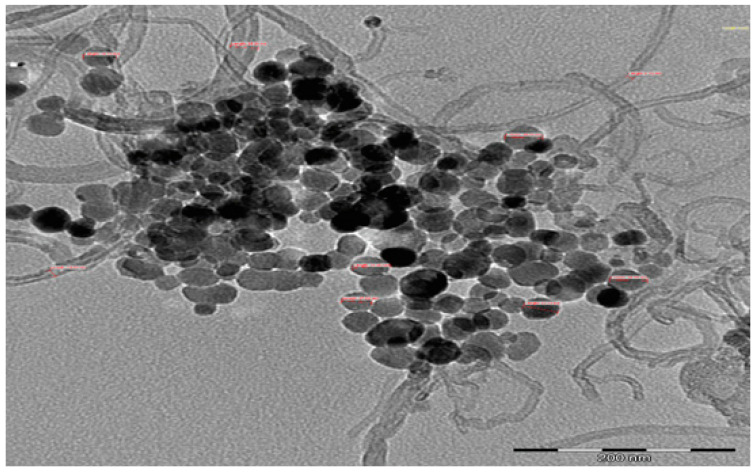
Morphology of the hybrid nanofluid using TEM.

**Figure 4:**
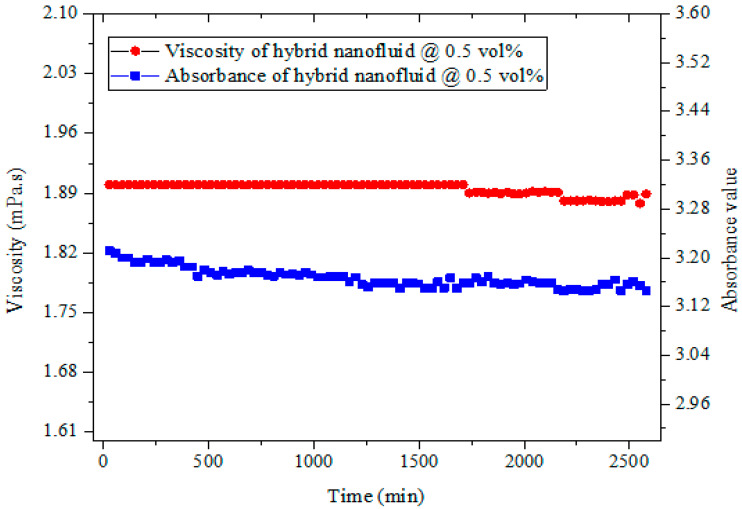
Stability monitoring of 0.5 vol% hybrid nanofluid using viscosity and absorbance.

**Figure 5:**
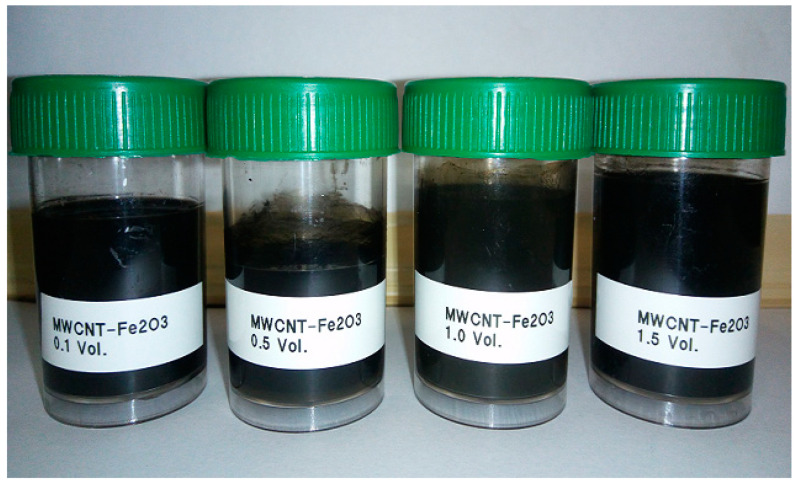
Visual stability of the hybrid nanofluids.

The morphology and dispersion of the hybrid nanoparticles are presented in Figure 3. A good suspension of the hybrid nanoparticles was observed. The UV–visible, viscosity, and visual methods were used to check the stability of the prepared hybrid nanofluids. As it is widely used for studying the stability of nanofluids [7,8], the absorbance of our hybrid nanofluids was measured using UV–visible spectrophotometry in order to assess their stability status. Figure 4 displays the viscosity and absorbance of the hybrid nanofluid (0.5 vol%) for 2580 min (43 h). The absorbance was around 3.2 with a peak wavelength of 261 nm, while the viscosity (at 15 °C) was around 2.0 mPas for the monitored period. These parameters (absorbance and viscosity) depicted the stability of the nanofluid, as a straight line relatively parallel to the horizontal was noticed for each parameter. An absorbance range of 3.0–3.8 with wavelengths of 287–264 nm was measured for the hybrid nanofluids at varying volume concentrations (0.1–1.5%). It can be noticed that the absorbance increased with a rise in the volume concentration, which was in agreement with previous studies [43,44]. It was observed that the increased suspension of the bi-nanoparticles into DIW altered the values of both the absorbance and the wavelength. A careful visual inspection of the samples (even titling the sample vial) was also done and no sedimentation was noticed after a month upon inspection (Figure 5). 

### 3.3. Electrical Conductivity of Hybrid Nanofluid

**Figure 6:**
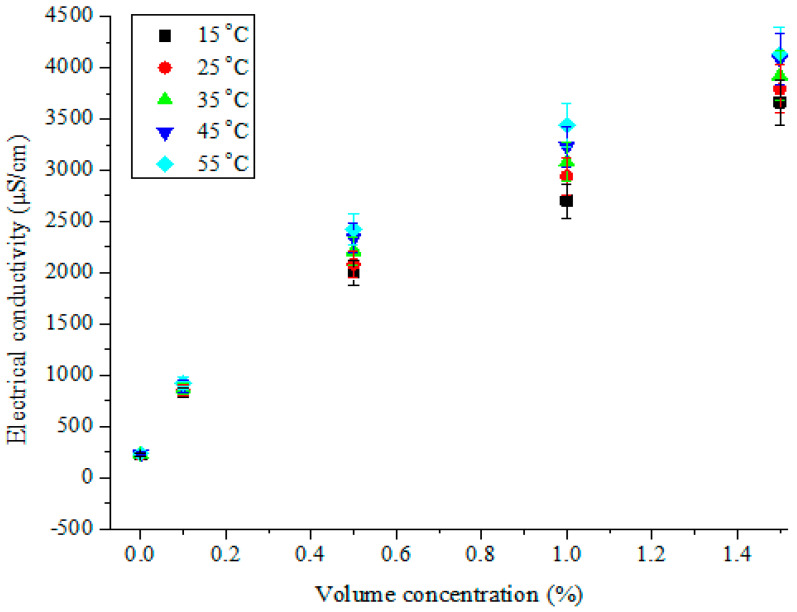
Electrical conductivity of hybrid nanofluids against volume concentration at different temperatures.

**Figure 7:**
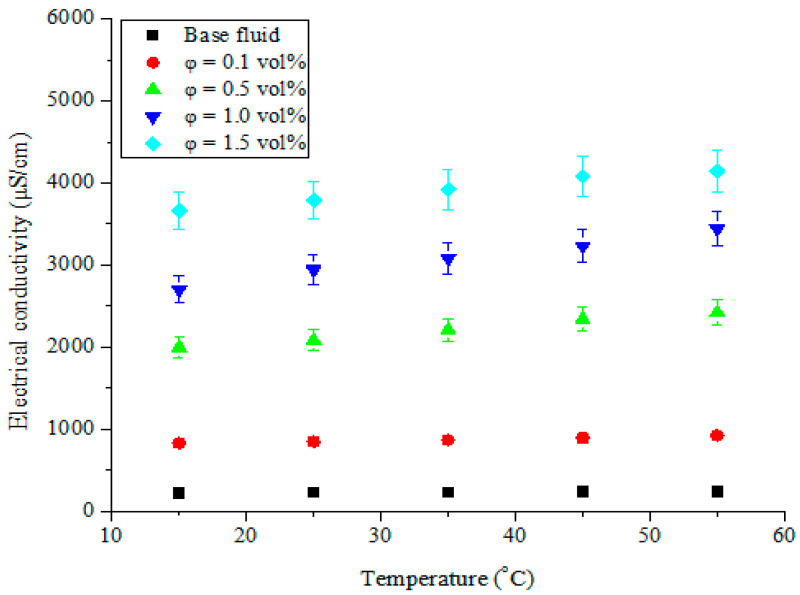
Electrical conductivity of hybrid nanofluids against temperature at different volume concentrations.

The capability of an aqueous solution to allow the passage of an electric current with the application of a potential difference has been termed “electrical conductivity”. The dispersion of nanoparticles into base fluids (having known electrical conductivity values) to form nanofluids resulted in improved electrical conductivity in the base fluids due to the increased presence and mobility of electric charges. The hybrid nanofluids of MWCNT-Fe_2_O_3_/DIW, as electrically conducting fluids, have been investigated for their electrical conductivity at varying temperatures and volume concentrations, as illustrated in Figure 6 and Figure 7, respectively. The electrical conductivity of MWCNT-Fe_2_O_3_/DIW nanofluids was enhanced significantly as the volume concentration increased (Figure 6). This can be linked to an increase in the electric charges in the MWCNT-Fe_2_O_3_/DIW nanofluids as the volume concentration rose. The electrical conductivity was noticed to be linearly dependent on the volume concentration of the nanofluids. Subject to a temperature increase, a slight enhancement of electrical conductivity was noticed (Figure 6). This observation is well illustrated in Figure 7, in which the electrical conductivity was improved linearly as the temperature increased. Consequently, the electrical conductivity of the nanofluids was directly proportional to the volume concentration and temperature, which was consistent with earlier studies reported in the literature for mono-particle and hybrid nanofluids [14,38,45,46,47,48]. At 55 °C and 1.5 vol%, maximum electrical conductivity (4139 μS/cm) was obtained which was considerably higher than the values of 19.0 μS/cm (for Fe_3_O_4_/water nanofluid at 0.6 vol% and 60 °C) and 1127–1265 μS/cm (Al_2_O_3_-MWCNT (80:20)/DIW nanofluid at 0.1 vol% and 50 °C) reported by Bagheli et al. [49] and Giwa et al. [38], respectively. However, Giwa et al. [48] published a range of 640–4570 μS/cm for Al_2_O_3_-Fe_2_O_3_ (75:25)/DIW nanofluid (at 0.75 vol% and 50 °C), which had a maximum value slightly higher than that reported for MWCNT-Fe_2_O_3_ (20:80)/DIW nanofluid in the present work. The electrical conductivity values were found to be strongly connected to the types of nanoparticles and the mixture ratios used in formulating the hybrid nanofluids.

**Figure 8:**
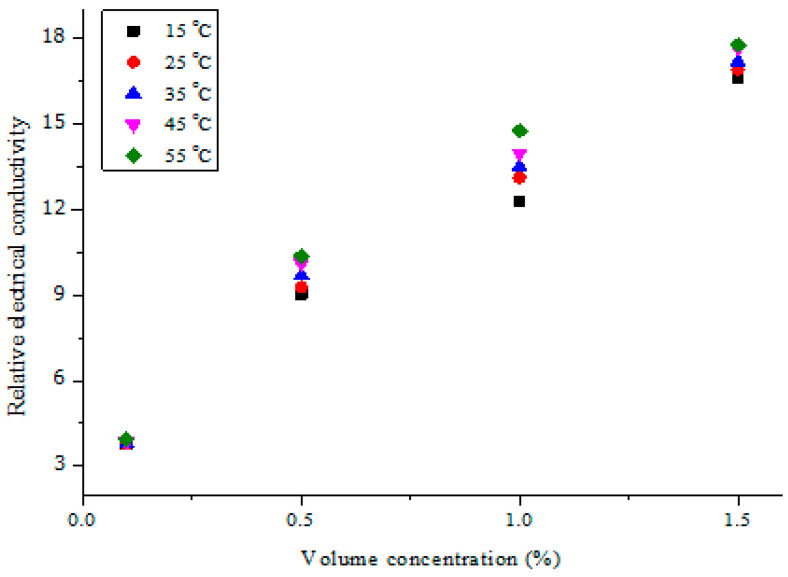
Relative electrical conductivity of hybrid nanofluids against volume concentration at different temperatures.

In Figure 8, the relative electrical conductivity of MWCNT-Fe_2_O_3_/DIW nanofluids at varying volume concentrations is presented. The continued suspension of Fe_2_O_3_ and MWCNT nanoparticles into DIW demonstrated a considerable increase in the relative electrical conductivity, while an increment in temperature slightly enhanced this property. The obtained result was in line with the work of Adio et al. [14], in which the relative electrical conductivity of MgO-EG nanofluids was enhanced with an increase in the nanofluid volume concentration. However, for their study, the temperature only increased relative electrical conductivity up to 30 °C, after which it declined. The obtained relative effective conductivity of MWCNT-Fe_2_O_3_/DIW nanofluids ranged from 3.95 to 17.76 for all the concentrations (vol.) at 55 °C.

**Figure 9:**
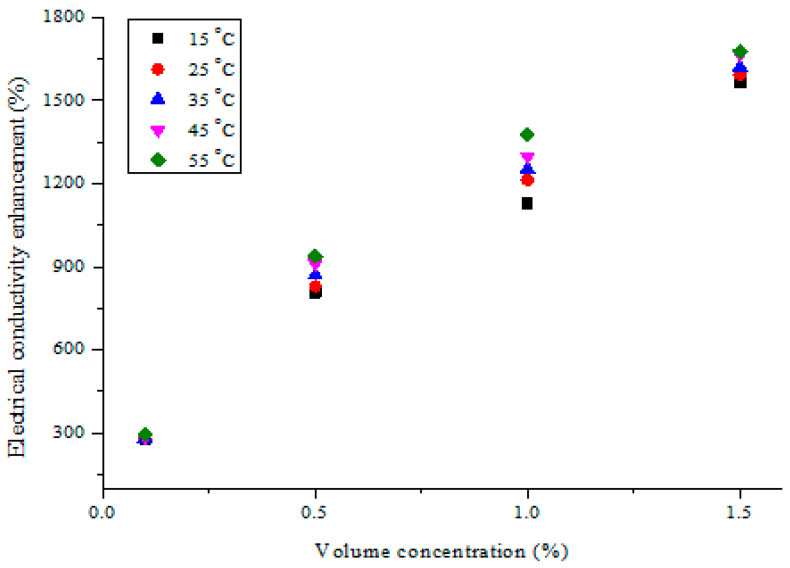
Electrical conductivity enhancement of hybrid nanofluids against volume concentration at different temperatures.

The electrical conductivity enhancement recorded by the addition of the hybrid nanoparticles to DIW at varying temperatures and volume concentrations is provided in Figure 9. In relation to DIW, the electrical conductivity of MWCNT-Fe_2_O_3_/DIW nanofluid was augmented significantly as the volume concentration rose but with a small enhancement as the temperature surged. It can be observed in Figure 9 that a relatively linear correlation existed between the enhancement of electrical conductivity and nanofluid volume concentration. A similar trend was noticed for the electrical conductivity enhancement with temperature, though with slight improvement. This result was found to agree with previous works on the electrical conductivity enhancement of nanofluids [44,47,50]. However, some studies reported either independence from temperature in the enhancement or a reduction in electrical conductivity with a temperature increase [43,47,51]. Maximum enhancement was recorded at 55 °C for all samples of the hybrid nanofluids. Therefore, at 55 °C and 1.5 vol%, an enhancement of 1676.4% was attained. 

In comparison to previous studies, Bagheli et al. [49] (Fe_3_O_4_/water nanofluid; 60 °C and 0.5 vol%), Sundar et al. [47] (nanodiamond-nickel/water nanofluid; 0.1 vol% and 65 °C), Kumar et al. [44] (MWCNT/water nanofluid; 0.6 vol% and 50 °C), Mehrali et al. [43] (nitrogen-doped graphene/water nanofluid; 60 °C and 0.06 wt%), Adio et al. [14] (EG-based MgO nanofluid; 0.5 vol% and 25 °C), Giwa et al. [38] (Al_2_O_3_-MWCNT (80:20)/DIW nanofluid; 0.1 vol% and 50 °C), and Giwa et al. [48] (Al_2_O_3_-Fe_2_O_3_ (75:25)/DIW nanofluid; 0.75 vol% and 50 °C) measured enhancements of 360%, 853.15%, 1814.96%, 190.57%, 6000%, 134.12–255.34%, and 163.37–1692.16%, respectively, for the electrical conductivity of different mono-particles and hybrid nanofluids. The nanofluid types, size of nanoparticles, volume/weight concentration or fraction, mixing ratio (for hybrid nanofluid), and temperature used in the various studies may be responsible for the variation in the results. However, a comparison of the works of Kumar et al. [44] and Bagheli et al. [49] with those of Giwa et al. [38] and Giwa et al. [48] supported the finding in the present study for MWCNT-Fe_2_O_3_/DIW nanofluid with regard to the augmentation of electrical conductivity because of the hybridization of bi-nanoparticles.

### 3.4. Viscosity of Hybrid Nanofluid

**Figure 10:**
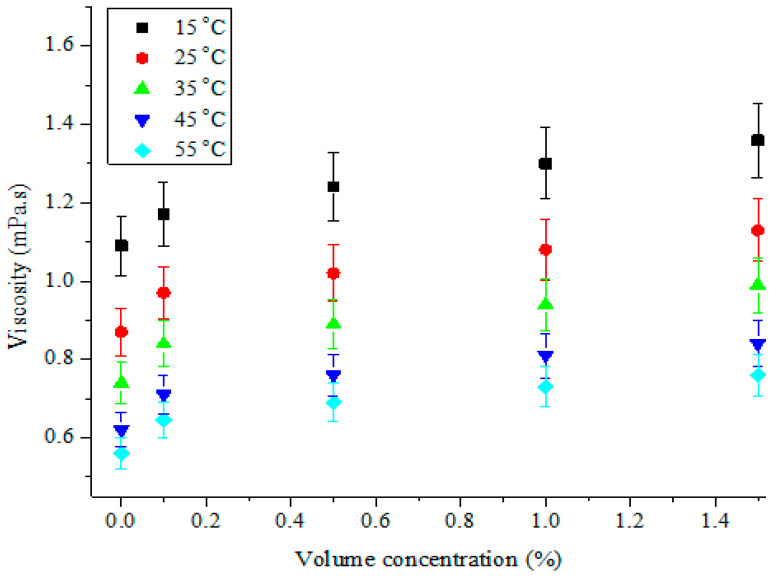
Viscosity of hybrid nanofluids against volume concentration at different temperatures.

**Figure 11:**
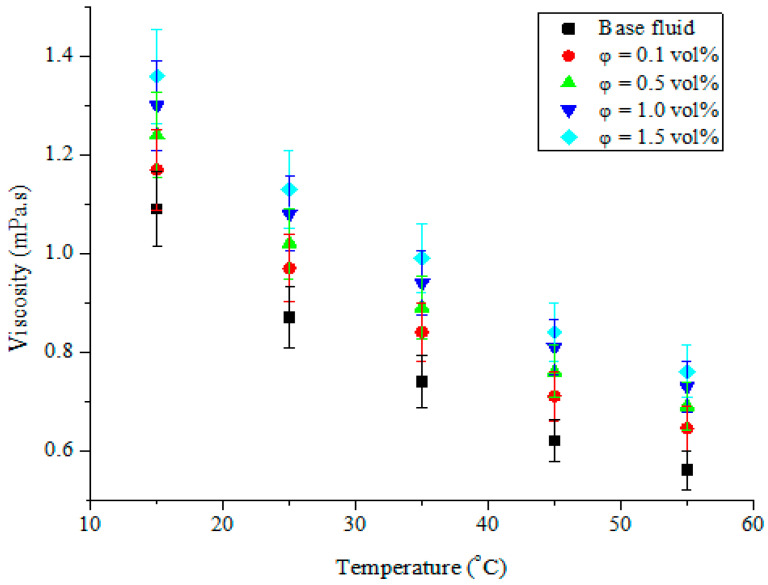
Viscosity of hybrid nanofluids against temperature at different volume concentrations.

An examination of the influence of the studied temperatures and volume concentrations on the viscosity of MWCNT-Fe_2_O_3_/DIW nanofluids was carried out. The viscosity of the hybrid nanofluids under varying volume concentrations and temperatures is shown in Figure 10 and Figure 11, respectively. An appreciation of the volume concentration of MWCNT-Fe_2_O_3_ (20:80)/DIW nanofluid was observed to enhance the viscosity in a linear pattern (Figure 10). This was because of the higher density of the hybrid nanoparticles in relation to DIW. An increment in the volume concentration due to the amount of hybrid nanoparticles suspended in DIW was noticed to enhance the viscosity of the nanofluid. Nanofluid viscosity was also observed to be dependent on the temperature, as depicted in Figure 11. The increasing change in the temperature was found to lessen the viscosity of the nanofluids. From Figure 10 and Figure 11, it can be observed that the influence of temperature on the viscosity of the hybrid nanofluids was more than that of volume concentration. Thus, the viscosity was dependent on both variables. The obtained results agreed with the works of Nadooshan et al. [34], Mehrali et al. [43], Adio et al. [45,52], Giwa et al. [38], Sharifpur et al. [53], and Giwa et al. [54], for the viscosity–temperature and viscosity–volume concentration relationships. In the present study, the viscosity of MWCNT-Fe_2_O_3_/DIW nanofluids ranged from 0.65 to 1.36 mPas for the ranges of volume concentration and temperature investigated. This was slightly higher than the range of 0.51 to 1.11 mPas and 0.57 to 1.13 mPas published by Gangadevi and Vinayagam [55] and Giwa et al. [48] for Al_2_O_3_-CuO/water nanofluid (0.2 vol% and at 20–60 °C) and Al_2_O_3_-Fe_2_O_3_/DIW nanofluid (0.75 vol% and at 20–50 °C).

**Figure 12:**
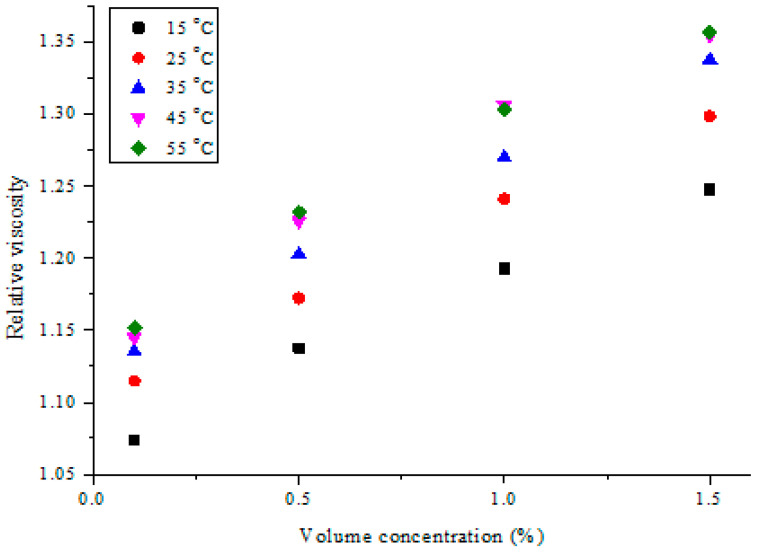
Relative viscosity of hybrid nanofluids against volume concentration at different temperatures.

As shown in Figure 12, the relative viscosity of MWCNT-Fe_2_O_3_/DIW nanofluids was compared with DIW for the studied volume concentrations. An increment in the concentration of the bi-nanoparticles was found to enhance the relative viscosity of the nanofluid. A seemingly linear correlation was observed between volume concentration and relative viscosity. In addition, the relative viscosity of MWCNT-Fe_2_O_3_/DIW nanofluids was noticed to increase as the temperature increased. The observed trend agreed with the data published by Nadooshan et al. [34] (Fe_3_O_4_-MWCNT/EG nanofluid), Adio et al. [45] (Al_2_O_3_-glycerol nanofluid), and Zawawi et al. [56] (hybrid nanofluids). However, Dardan et al. [26] reported the reverse of the trend noticed in the present work, as the relative viscosity of Al_2_O_3_-MWCNT/EO nanofluid was reduced with temperatures at 35–50 °C, for measurements spanning 25–50 °C. At 55 °C, the relative viscosity of the nanofluids examined in this work increased from 1.152 (0.1 vol%) to 1.357 (1.5 vol%). 

**Figure 13:**
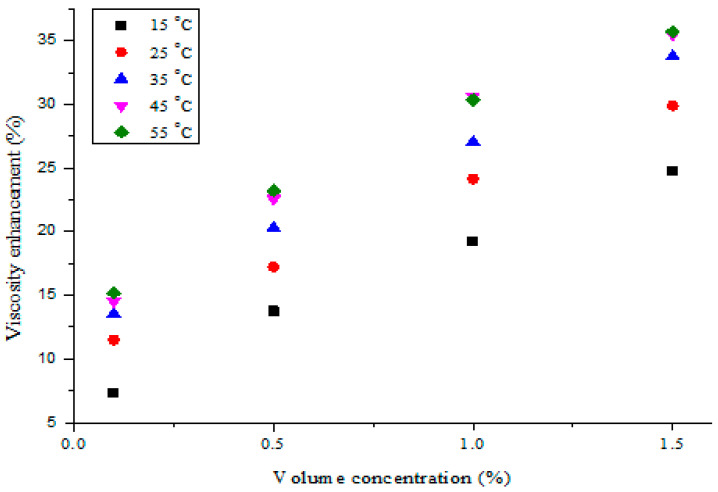
Viscosity enhancement of hybrid nanofluids against volume concentration at different temperatures.

The percentage enhancement of the hybrid nanofluid viscosity compared with DIW under varying temperatures and volume concentrations is illustrated in Figure 13. It can be noticed that a rise in the volume concentration of MWCNT-Fe_2_O_3_ (20:80)/DIW nanofluids resulted in substantial viscosity enhancement when compared with DIW. Relatively linear enhancement of the viscosity was noticed for MWCNT-Fe_2_O_3_ (20:80)/DIW nanofluids, as the concentration and temperature rose when compared with DIW. For this work, the highest viscosity enhancement of 35.7% was estimated for the hybrid nanofluids compared to DIW. Previous studies have recorded viscosity enhancements of 58% (MWCNT; 1 vol%) [57], 20.5% (Al_2_O_3_-TiO_2_/PAG; 0.1 vol%) [56], 43.52% (MWCNT-CuO/EO; 1 vol%) [58], 46% (Al_2_O_3_-MWCNT/EO; 1.0 vol%) [26], 24.56% (Al_2_O_3_-MWCNT/DIW; 0.1 vol%) [38], 20% (ZnO-MWCNT/EO; 0.8 vol%) [37], 36.4% (CuO-MgO-TiO_2_/DIW; 0.5 vol%) [36], and 43.64% (Al_2_O_3_-Fe_2_O_3_/DIW; 0.75 vol%) [48] for mono-particle and hybrid nanofluids, when compared with the respective base fluids. The results from the present work showed a relatively lower viscosity enhancement in relation to earlier studies, which can be attributed to the types of hybrid nanoparticles and base fluids utilized in preparing MWCNT-Fe_2_O_3_ (20:80)/DIW nanofluids. MWCNT nanoparticles are known to have significantly lower density in comparison to metal oxide-based nanoparticles.

### 3.5. Correlation Development

Formulated classical models and theoretical correlations for estimating various thermal properties of different nanofluids have been demonstrated by numerous studies to be inadequate for their estimation [20,36,51,53,59]. They are found to either underestimate or overestimate the measured data of the thermal properties of nanofluids. Research progress showed that there is a need to formulate correlations to effectively estimate the thermal properties of MWCNT-Fe_2_O_3_ (20:80)/DIW nanofluid. The uniqueness of the physicochemical properties of the diverse nanoparticles and base fluids used to prepare hybrid nanofluids and the fitting of the experimental data to estimate the thermal properties are now becoming more important and relevant.

Curve fittings of the experimental data of the relative electrical conductivity and relative viscosity garnered for MWCNT-Fe_2_O_3_ (20:80)/DIW nanofluids were performed. The formulas developed from the data of relative electrical conductivity and viscosity as dependencies of temperature and volume concentration are expressed in Equations (8) and (9), respectively. For the electrical conductivity correlation, R^2^ = 0.968, coefficient of corelation (R) = 0.984, root mean square error (RMSE) = 0.16, and mean absolute percentage error (MAPE) = 11.085, while for the viscosity correlation (linear regression), R^2^ = 0.966, R = 0.983, RMSE = 0.166, and MAPE = 8.721. These variables revealed relatively high coefficients of determination and correlation coefficients with significantly low errors for both correlations.

(8) $$\frac{\sigma_{hnf}}{\sigma_{bf}}=2.757+0.032T+9.287\varphi$$

(9) $$\frac{\mu_{hnf}}{\mu_{bf}}=1.031+0.0025T+0.1386\varphi$$

**Figure 14:**
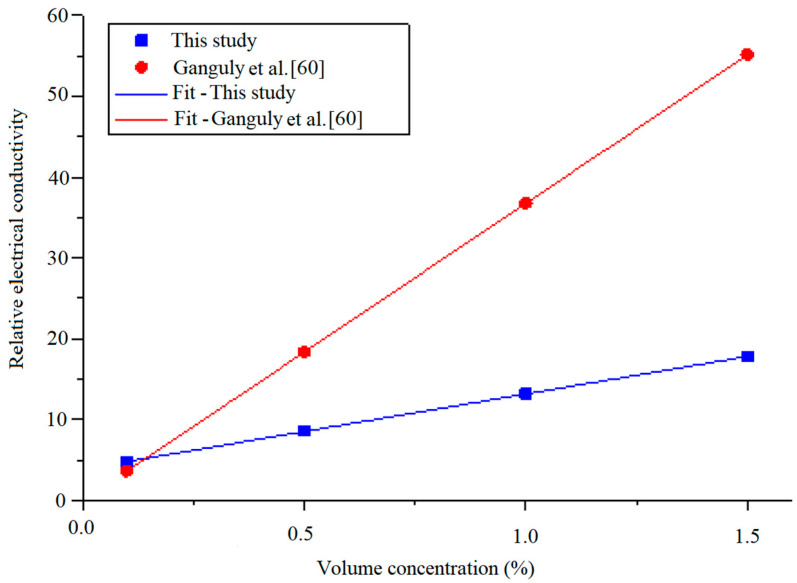
Developed correlations for electrical conductivity compared to an existing correlation at different temperatures.

**Figure 15:**
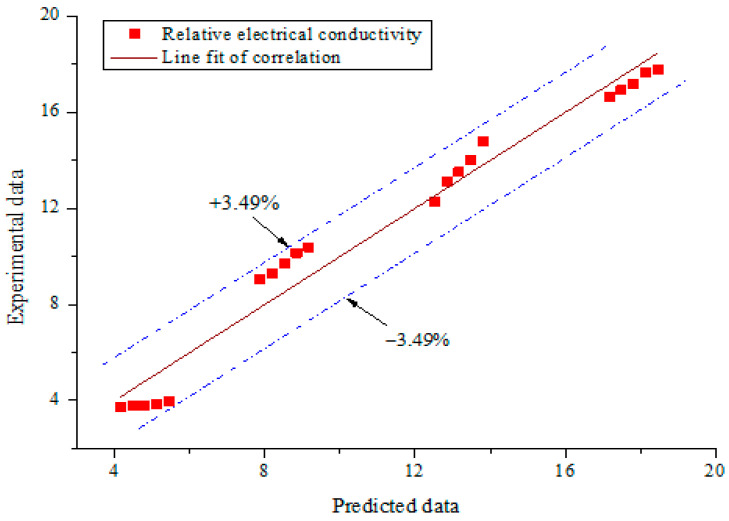
Correlation of experimental and predicted data (electrical conductivity).

The correlation from the work of Ganguly et al. [60] and that derived using Equation (8) for the relative electrical conductivity of MWCNT-Fe_2_O_3_ (20:80)/DIW at 35 °C are plotted in Figure 14. It is obvious that the existing correlation, as published in the literature, overestimated the experimental data of the electrical conductivity of MWCNT-Fe_2_O_3_ (20:80)/DIW nanofluid. This was because the existing correlation was formulated using data from a different nanofluid (Al_2_O_3_/water) to that utilized (MWCNT-Fe_2_O_3_/water) in this study, hence, it could not adequately predict the property. The correlation relating the experimental and predicted data of the relative electrical conductivity of MWCNT-Fe_2_O_3_ (20:80)/DIW nanofluid is presented in Figure 15. A straight line was noticed to relate the predicted and experimental values, showing a good correlation between both data sets. The MOD for the correlation was ±3.48%. 

**Figure 16:**
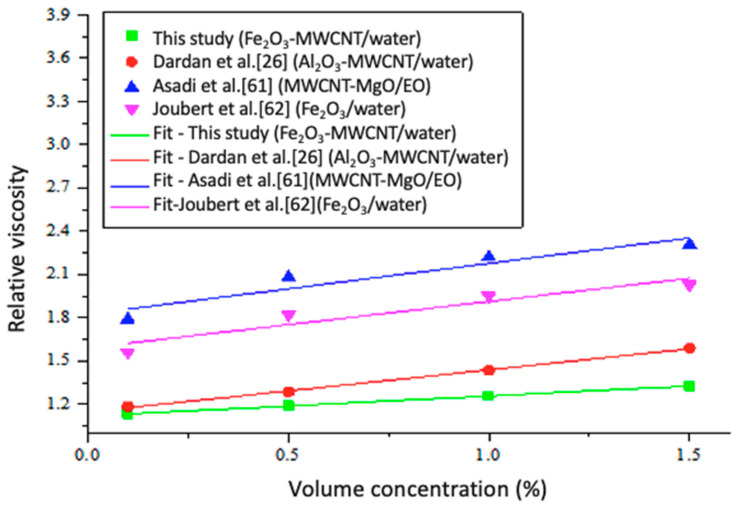
Developed correlations for viscosity compared to existing correlations at different temperatures.

**Figure 17:**
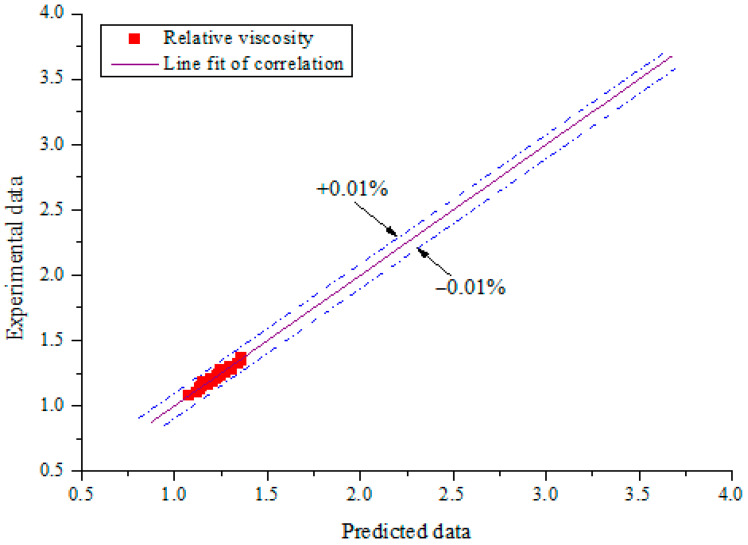
Correlation of experimental and predicted data (viscosity).

A plot of the fitted relative viscosity correlation for this work and those formulated in previous studies at 35 °C is presented in Figure 16 [26,61,62]. It shows that none of the existing correlations for estimating the viscosity of mono-particle and hybrid nanofluids could fit the obtained experimental data. They all overestimated the relative viscosity of the investigated MWCNT-Fe_2_O_3_/DIW nanofluid. The obtained relative viscosity of MWCNT-Fe_2_O_3_/DIW nanofluid was overestimated using the experiment-derived correlation for Fe_2_O_3_/DIW nanofluid [62]. This revealed a reduction (49.8%) in the viscosity of MWCNT-Fe_2_O_3_ (20:80)/DIW nanofluid because of the hybridization of Fe_2_O_3_ nanoparticles with MWCNT nanoparticles. With the evident reduction in the viscosity of MWCNT-Fe_2_O_3_/DIW nanofluid, the use of this hybrid nanofluid is favorable for engineering applications in terms of lower pumping power. A linear relationship was found to occur between the experimental and predicted data, as presented in Figure 17. An MOD of ±0.01% was found.

## 4. Conclusions

Stable MWCNT-Fe_2_O_3_/DIW nanofluids have been prepared and investigated for their electrical conductivity and viscosity at varying temperatures and volume concentrations. Increasing the bi-nanoparticles’ concentration enhanced both the viscosity and electrical conductivity of MWCNT-Fe_2_O_3_/DIW nanofluids. Additionally, increasing the nanofluid temperature augmented the electrical conductivity, whereas the viscosity was reduced. Relative to the base fluid, maximum enhancements of 1676.4% and 35.7% were achieved for the electrical conductivity and viscosity of MWCNT-Fe_2_O_3_/DIW nanofluids, respectively. The obtained results were noticed to be consistent with previous studies in the literature. The hybridization of MWCNT and Fe_2_O_3_ nanoparticles to prepare MWCNT-Fe_2_O_3_ (20:80)/DIW nanofluid was proven to cause a reduction of viscosity, which is advantageous in engineering applications of the fluid. To predict the viscosity and electrical conductivity of the studied MWCNT-Fe_2_O_3_/DIW nanofluids, correlations have been developed. 

## Figures and Tables

**Table 1:** Thermophysical properties of studied materials at room temperature.

Properties	Deionized Water [38]	Multi-Walled Carbon Nanotube [38]	γ-Fe_2_O_3_ [39,40]
Density (kg/m^3^)	997	2100	5242
Thermal conductivity (W/m·K)	0.613	2000	20
Heat capacity (J/kg·K)	4179	710	681

## References

[1] Masuda H., Ebata A., Teramae K., Hishinuma N. (1993). Alteration of thermal conductivity and viscosity of liquid by dispersing ultra-fine particles. Netsu. Bussei..

[2] Eastman J.A., Choi S.U.S., Li S., Yu W., Thompson L.J. (2001). Anomalously increased effective thermal conductivities of ethylene glycol-based nanofluids containing copper nanoparticles. Appl. Phys. Lett..

[3] Murshed S.M.S., Leong K.C., Yang C. (2008). Investigations of Thermal Conductivity and Viscosity of Nanofluids. Int. J. Therm. Sci..

[4] Murshed S.M.S., Leong K.C., Yang C. (2008). Thermophysical and Electrokinetic Properties of Nanofluids—A Critical Review. Appl. Therm. Eng..

[5] Prasher R., Song D., Wang J., Phelan P. (2006). Measurements of nanofluid viscosity and its implications for thermal applications. Appl. Phys. Lett..

[6] Abareshi M., Sajjadi S.H., Zebarjad S.M., Goharshadi E.K. (2011). Fabrication, characterization, and measurement of viscosity of α-Fe_2_O_3_-glycerol nanofluids. J. Mol. Liq..

[7] Murshed S.M.S., Nieto de Castro C.A. (2014). Nanofluids: Synthesis, Properties and Applications.

[8] Cacua K., Murshed S.M.S., Pabón E., Buitrago R. (2020). Dispersion and thermal conductivity of TiO_2_/water nanofluid. J. Therm. Ana. Calor..

[9] Namburu P.K., Kulkarni D.P., Misra D., Das D.K. (2007). Viscosity of copper oxide nanoparticles dispersed in ethylene glycol and water mixture. Exp. Therm. Fluid Sci..

[10] Murshed S.M.S., Estell P. (2017). A State of the Art Review on viscosity of nanofluids. Renew. Sustain. Energy Rev..

[11] Murshed S.M.S., Nieto de Castro C.A. (2011). Contribution of Brownian motion in thermal conductivity of nanofluids. Proc. World Cong. Eng..

[12] Murshed S.M.S. (2009). Correction and comment on “thermal conductance of nanofluids: Is the controversy over?”. J. Nanopart. Res..

[13] Murshed S.M.S., Nieto de Castro C.A. (2012). Predicting the thermal conductivity of nanofluids-Effect of Brownian motion of nanoparticles. J. Nanofluid..

[14] Adio S.A., Sharifpur M., Meyer J.P. (2015). Factors affecting the pH and electrical conductivity of MgO-ethylene glycol nanofluids. Bull. Mater. Sci..

[15] Sharifpur M., Yousefi S., Meyer J.P. (2016). A new model for density of nanofluids including nanolayer. Int. Commun. Heat Mass Transf..

[16] Nabati Shoghl S., Jamali J., Keshavarz Moraveji M. (2016). Electrical conductivity, viscosity, and density of different nanofluids: An experimental study. Exp. Therm. Fluid Sci..

[17] Nieto de Castro C.A., Murshed S.M.S., Lourenço M.J.V., Santos F.J.V., Lopes M.L.M., França J.M.P. (2012). Enhanced thermal conductivity and specific heat capacity of carbon nanotubes ionanofluids. Int. J. Therm. Sci..

[18] Fal J., Barylyak A., Besaha K., Bobitski Y.V., Cholewa M., Zawlik I., Szmuc K., Cebulski J. (2016). Experimental investigation of electrical conductivity and permittivity of SC-TiO_2_-EG nanofluids. Nanoscale Res. Lett..

[19] Nor S., Azis N., Jasni J., Kadir M., Yunus R., Yaakub Z. (2017). Investigation on the electrical properties of palm oil and coconut oil based TiO_2_ nanofluids. IEEE Trans. Dielectr. Electr. Insul..

[20] Abdolbaqi M.K., Azmi W.H., Mamat R., Sharma K.V., Najafi G. (2016). Experimental investigation of thermal conductivity and electrical conductivity of bioglycol—Water mixture based Al_2_O_3_ nanofluid. Appl. Therm. Eng..

[21] Jana S., Salehi-Khojin A., Zhong W.H. (2007). Enhancement of fluid thermal conductivity by the addition of single and hybrid nano-additives. Thermochim. Acta..

[22] Suresh S., Venkitaraj K.P., Selvakumar P., Chandrasekar M. (2011). Synthesis of Al_2_O_3_-Cu/water hybrid nanofluids using two step method and its thermo physical properties. Colloids Surf. A Physicochem. Eng. Asp..

[23] Chen L., Yu W., Xie H. (2012). Enhanced thermal conductivity of nanofluids containing Ag/MWNT composites. Powder Technol..

[24] Abbasi S.M., Rashidi A., Nemati A., Arzani K. (2013). The effect of functionalisation method on the stability and the thermal conductivity of nanofluid hybrids of carbon nanotubes/gamma alumina. Ceram Int..

[25] Hemmat Esfe M., Saedodin S., Yan W.M., Afrand M., Sina N. (2016). Erratumto: Study on thermal conductivity of water-based nanofluids with hybrid suspensions of CNTs/Al_2_O_3_ nanoparticles. J. Therm. Anal. Calorim..

[26] Dardan E., Afrand M., Meghdadi Isfahani A.H. (2016). Effect of suspending hybrid nano-additives on rheological behavior of engine oil and pumping power. Appl. Therm. Eng..

[27] Afrand M., Nazari Najafabadi K., Akbari M. (2016). Effects of temperature and solid volume fraction on viscosity of SiO_2_-MWCNTs/SAE40 hybrid nanofluid as a coolant and lubricant in heat engines. Appl. Therm. Eng..

[28] Megatif L., Ghozatloo A., Arimi A., Shariati-Niasar M. (2016). Investigation of laminar convective heat transfer of a novel TiO_2_-carbon nanotube hybrid water-based nanofluid. Exp. Heat Transf..

[29] Eshgarf H., Afrand M. (2016). An experimental study on rheological behavior of non-Newtonian hybrid nano-coolant for application in cooling and heating systems. Exp. Therm. Fluid Sci..

[30] Kakavandi A., Akbari M. (2018). Experimental investigation of thermal conductivity of nanofluids containing of hybrid nanoparticles suspended in binary base fluids and propose a new correlation. Int. J. Heat Mass Transf..

[31] Esfe M.H., Esfandeh S., Afrand M., Rejvani M., Rostamian S.H. (2018). Experimental evaluation, new correlation proposing and ANN modeling of thermal properties of EG based hybrid nanofluid containing ZnO-DWCNT nanoparticles for internal combustion engines applications. Appl. Therm. Eng..

[32] Moldoveanu G.M., Minea A.A., Huminic G., Huminic A. (2019). Al_2_O_3_/TiO_2_ hybrid nanofluids thermal conductivity: An experimental approach. J. Therm. Anal. Calorim..

[33] Harandi S.S., Karimipour A., Afrand M., Akbari M., D’Orazio A. (2016). An experimental study on thermal conductivity of F-MWCNTs-Fe_3_O_4_/EG hybrid nanofluid: Effects of temperature and concentration. Int. Commun. Heat Mass Transf..

[34] Nadooshan A.A., Eshgarf H., Afrand M. (2018). Measuring the viscosity of Fe_3_O_4_-MWCNTs/EG hybrid nanofluid for evaluation of thermal efficiency: Newtonian and non-Newtonian behavior. J. Mol. Liq..

[35] Shi L., He Y., Hu Y., Wang X. (2018). Thermophysical properties of Fe_3_O_4_@CNT nanofluid and controllable heat transfer performance under magnetic field. Energy Convers. Manag..

[36] Mousavi S.M., Esmaeilzadeh F., Wang X.P. (2019). Effects of temperature and particles volume concentration on the thermophysical properties and the rheological behavior of CuO/MgO/TiO_2_ aqueous ternary hybrid nanofluid Experimental investigation. J. Therm. Anal. Calorim..

[37] Goodarzi M., Toghraie D., Reiszadeh M., Afrand M. (2019). Experimental evaluation of dynamic viscosity of ZnO–MWCNTs/engine oil hybrid nanolubricant based on changes in temperature and concentration. J. Therm. Anal. Calorim..

[38] Giwa S.O., Sharifpur M., Meyer J.P. (2020). Experimental study of thermo-convection performance of hybrid nanofluids of Al_2_O_3_-MWCNT/water in a differentially heated square cavity. Int. J. Heat Mass Transf..

[39] Mussatti EMerlini C., de Oliveira Barra G.M., Güths S., de Oliveira A.P.N., Siligardi C. (2013). Evaluation of the properties of Iron oxide-filled castor oil polyurethane. Mater. Res..

[40] Korolev V.V., Arefyev I.M., Blinov A.V. (2008). Heat capacity of superfine oxides of iron under applied magnetic fields. J. Therm. Anal. Calorim..

[41] Sharker K.K., Islam M.N., Das S. (2017). Counterion effect on Krafft temperature and related properties of octadecyltrimethylammonium bromide. J. Surfactants Deterg..

[42] Topallar H., Karadag B. (1998). Mechanism of micelle formation in sodium dodecyl sulfate and cetyltrimethylammonium bromide. J. Surfactants Deterg..

[43] Mehrali M., Sadeghinezhad E., Latibari S.T., Mehrali M., Togun H., Zubir M.N.M., Kazi S.N., Metselaar H.S.C. (2014). Preparation, characterization, viscosity, and thermal conductivity of nitrogen-doped graphene aqueous nanofluids. J. Mater. Sci..

[44] Kumar P.G., Kumaresan V., Velraj R. (2017). Stability, viscosity, thermal conductivity, and electrical conductivity enhancement of multi-walled carbon nanotube nanofluid using gum arabic. Fuller. Nanotub. Carbon Nanostruct..

[45] Adio S.A., Sharifpur M., Meyer J.P. (2015). Investigation into effective viscosity, electrical conductivity, and pH of γ-Al_2_O_3_-glycerol nanofluids in Einstein concentration regime. Heat Transf. Eng..

[46] Said Z., Allagui A., Abdelkareem M.A., Alawadhi H., Elsaid K. (2018). Acid-functionalized carbon nanofibers for high stability, thermoelectrical and electrochemical properties of nanofluids. J. Colloid Interface Sci..

[47] Sundar L.S., Shusmitha K., Singh M.K., Sousa A.C.M. (2014). Electrical conductivity enhancement of nanodiamond-nickel (ND-Ni) nanocomposite based magnetic nanofluids. Int. Commun. Heat Mass Transf..

[48] Giwa S.O., Sharifpur M., Goodarzi M., Alsulami H., Meyer J.P. (2020). Influence of base fluid, temperature, and concentration on the thermophysical properties of hybrid nanofluids of alumina—Ferrofluid: Experimental data, modeling through enhanced ANN, ANFIS, and curve fitting. J. Therm. Anal. Calorim..

[49] Bagheli S., Fadafan H.K., Orimi R.L., Ghaemi M. (2015). Synthesis and experimental investigation of the electrical conductivity of water based magnetite nanofluids. Powder Technol..

[50] Minea A.A., Luciu R.S. (2012). Investigations on electrical conductivity of stabilized water based Al_2_O_3_ nanofluids. Microfluid. Nanofluid..

[51] Khdher A.M., Sidik N.A.C., Hamzah W.A.W., Mamat R. (2016). An experimental determination of thermal conductivity and electrical conductivity of bio glycol based Al_2_O_3_ nanofluids and development of new correlation. Int. Commun. Heat Mass Transf..

[52] Adio S.A., Sharifpur M., Meyer J.P. (2016). Influence of ultrasonication energy on the dispersion consistency of Al_2_O_3_–glycerol nanofluid based on viscosity data, and model development for the required ultrasonication energy density. J. Exp. Nanosci..

[53] Sharifpur M., Adio S.A., Meyer J.P. (2015). Experimental investigation and model development for effective viscosity of Al_2_O_3_-glycerol nanofluids by using dimensional analysis and GMDH-NN methods. Int. Commun. Heat Mass Transf..

[54] Giwa S.O., Sharifpur M., Meyer J.P. (2020). Effects of uniform magnetic induction on heat transfer performance of aqueous hybrid ferrofluid in a rectangular cavity. Appl. Therm. Eng..

[55] Gangadevi R., Vinayagam B.K. (2019). Experimental determination of thermal conductivity and viscosity of different nanofluids and its effect on a hybrid solar collector. J. Therm. Anal. Calorim..

[56] Zawawi N.N.M., Azmi W.H., Redhwan A.A.M., Sharif M.Z., Samykano M. (2018). Experimental investigation on thermo-physical properties of metal oxide composite nanolubricants. Int. J. Refrig..

[57] Garbadeen I.D., Sharifpur M., Slabber J.M., Meyer J.P. (2017). Experimental study on natural convection of MWCNT-water nanofluids in a square enclosure. Int. Commun. Heat Mass Transf..

[58] Hemmat Esfe M., Sarlak M.R. (2017). Experimental investigation of switchable behavior of CuO-MWCNT (85–15%)/10W-40 hybrid nano-lubricants for applications in internal combustion engines. J. Mol. Liq..

[59] Saeedi A.H., Akbari M., Toghraie D. (2018). An experimental study on rheological behavior of a nanofluid containing oxide nanoparticle and proposing a new correlation. Phys. E Low Dimens. Syst. Nanostruct..

[60] Ganguly S., Sikdar S., Basu S. (2009). Experimental investigation of the effective electrical conductivity of aluminum oxide nanofluids. Powder Technol..

[61] Asadi A., Asadi M., Rezaei M., Siahmargoi M., Asadi F. (2016). The effect of temperature and solid concentration on dynamic viscosity of MWCNT/MgO (20–80)–SAE50 hybrid nano-lubricant and proposing a new correlation: An experimental study. Int. Commun. Heat Mass Transf..

[62] Joubert J.C., Sharifpur M., Solomon A.B., Meyer J.P. (2017). Enhancement in heat transfer of a ferrofluid in a differentially heated square cavity through the use of permanent magnets. J. Magn. Magn. Mater..

